# Analysis of mortality risk and influencing factors in female patients with young-onset type 2 diabetes

**DOI:** 10.3389/fendo.2026.1735053

**Published:** 2026-04-17

**Authors:** Ling Xiang, Mingmin Zhu, Zi Yan, Zhijin Geng, Yuankai Zhou, Zhongming Sun, Dandan Miao, Yong Zhang, Jinyi Zhou, Enchun Pan, Wen Hu

**Affiliations:** 1 The Affiliated Huai’an Hospital of Xuzhou Medical University/The Second People’s Hospital of Huai’an, Huai’an, Jiangsu, China; 2 Department of Endocrinology, Sheyang County People’s Hospital, Yancheng, Jiangsu, China; 3 Huai‘an Center for Disease Control and Prevention, Huai‘an, Jiangsu, China; 4 Jiangsu Provincial Center for Disease Control and Prevention, Nanjing, China

**Keywords:** all-cause mortality, Chinese patients, risk factors, TyG-BMI, young-onset type 2 diabetes mellitus, female patients

## Abstract

**Objective:**

To explore all-cause mortality risk and its influencing factors in female patients with young-onset type 2 diabetes mellitus (YOD).

**Methods:**

This cohort study included 5,984 female patients diagnosed with type 2 diabetes mellitus (T2DM) who were registered in the National Basic Public Health Service Management Program between December 2013 and January 2014 in Qinghe District (now Qingjiangpu District) and Huai’an District, Huai’an City, Jiangsu Province, China. All-cause mortality data were obtained by comprehensive matching with the Huai’an city Resident Mortality Database as of December 31, 2024. All patients were divided into four groups according to age of onset and menopause status: the young menopausal group (YM), the young premenopausal group (YP), the nonyoung menopausal group (NYM), and the nonyoung premenopausal group (NYP). Multivariate Cox regression analysis was used to calculate hazard ratios (HRs) and corresponding 95% confidence intervals (95% CIs) for all-cause mortality risk during the follow-up period.

**Results:**

1) A total of 5984 T2DM patients, comprising 387 YOD patients and 5597 late-onset T2DM patients (LODs), with an average age of 61.77 ± 9.87 years, were included. During the ten-year follow-up, 1293 deaths were recorded, with an all-cause mortality rate of 21.6%. The mortality rate of YOD was lower than that of LOD. 2) Subgroup analysis revealed that NYM had the highest all-cause mortality rate at 24%, followed by YM at 15%, and YP had the lowest mortality rate at 3.7%. The cumulative survival curve indicated that the YM group had the lowest survival curve, followed by the NYM group, and the YP group had the highest survival curve. 3) Multivariate Cox regression analysis revealed that the triglyceride–glucose–body mass index (TyG-BMI) was an independent protective factor for the YM group (HR = 0.980, 95% CI = 0.965–0.997) (P = 0.018). For every one standard deviation increase in the TyG-BMI, the mortality risk decreased by 2%. UA was a common risk factor for all-cause mortality in women with different ages of onset before and after menopause.

**Conclusion:**

In this study, the overall all-cause mortality rate for YOD patients was lower than that for LOD patients. However, postmenopausal women with YOD have greater all-cause mortality than premenopausal women with LOD do, and their risk of death decreases with increasing TyG-BMI. Therefore, early identification and intervention of TyG-BMI levels in postmenopausal women with YOD may help identify high-risk individuals and guide early intervention.

## 1 Introduction

With global economic development and the increasing prevalence of obesity, the incidence of young-onset type 2 diabetes mellitus (YOD) has been increasing in various countries. In China, the prevalence has quadrupled over 13 years (1997–2010), whereas in Southeast Asia and the UK, it has increased tenfold and fivefold over 15 years (1991–2006), respectively, and in the U.S., it has risen by 31% over 9 years (2001–2009) (1–3).YOD is defined as type 2 diabetes mellitus (T2DM) with an onset age of less than 40 years (1). Compared with T2DM patients with an onset age of 40 years or older (late-onset type 2 diabetes (LOD), YOD patients have worse pancreatic function, higher risks of macrovascular complications and mortality, and are more prone to microvascular complications (4). A meta-analysis showed that each one-year delay in diabetes diagnosis was associated with a 3% reduction in macrovascular complications, a 5% reduction in microvascular complications, and a 4% decrease in all-cause mortality (5).

In recent years, the prevalence of type 2 diabetes among young women (typically aged 15–39) has been on the rise globally. According to data from the Global Burden of Disease Study (GBD) (6), between 1990 and 2021, the incidence of type 2 diabetes in adolescents and young women aged 10–24 increased significantly. The 2021 incidence rate was more than twice that of 1990, with an annual percentage change (AAPC) of 3.01%. Furthermore, the growth rate of diabetes-related mortality among women was nearly four times that of men, indicating a faster increase in the risk of death from diabetes for women. In China, the incidence of type 2 diabetes among young women is also rising. Studies (7) show that between 1990 and 2021, the average annual percentage change (AAPC) in female incidence was 2.59% (95% CI: 2.51%-2.66%, P < 0.001); the AAPC for DALYs was 2.63% (95% CI: 2.52%-2.74%, P < 0.001).

YOD places a significant burden on individuals and healthcare systems. However, research on the epidemiology, complications, and mortality risk of YOD patients in the Chinese population, particularly women, is limited. Therefore, it is necessary to conduct an in-depth investigation into the risk factors associated with mortality in female patients with YOD. This study utilized ten years of follow-up data from the “Huai’an Community-Based T2DM Patient Cohort Study” to analyze the epidemiology, complications, and mortality risk associations in female patients with YOD before and after menopause, aiming to provide epidemiological evidence for the clinical diagnosis and treatment of female patients with YOD, improve their quality of life, and reduce their mortality risk.

## 2 Methods

### 2.1 Ethics statement

The study was reviewed by the Ethics Committee of the Jiangsu Provincial CDC (approval number: 2013026), and all respondents signed an informed consent form before the formal investigation.

### 2.2 Study population

The inclusion criteria encompassed a total of 9759 patients diagnosed with T2DM who were duly registered and enrolled in the National Basic Public Health Service Management Program. This study specifically focused on patients residing in Qinghe District (now Qingjiangpu District) and Huai’an District, located in Huai’an City, Jiangsu Province, China, during the period spanning from December 2013 to January 2014.

Inclusion criteria: Female patients in T2DM registered and managed under the National Basic Public Health Services in the Qinghe District (now Qingjiangpu District) and Huai’an District of Huai’an City, Jiangsu Province, from December 2013 to January 2014.

**Figure 1:**
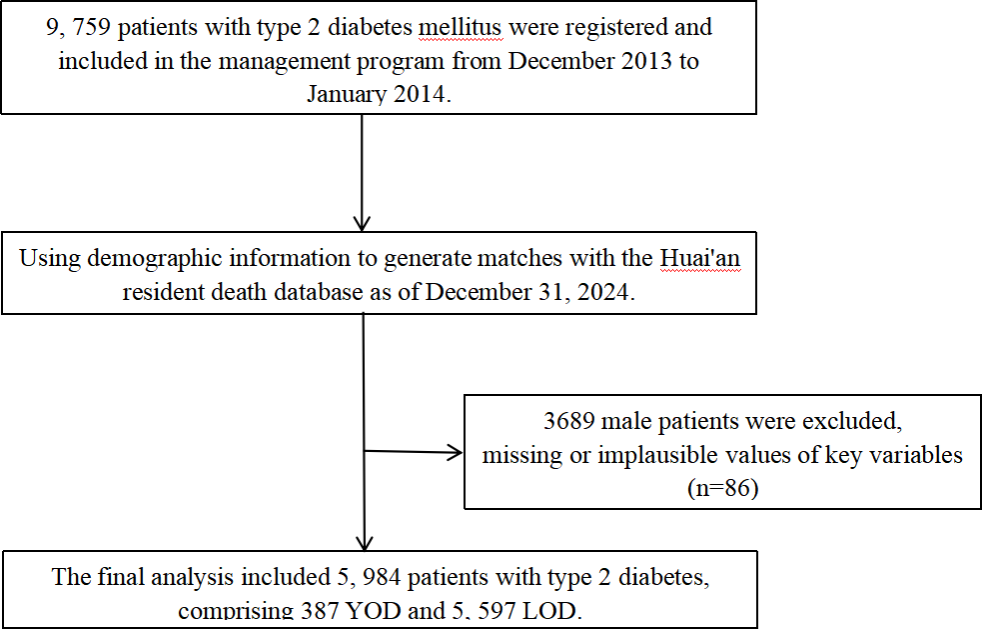
The process of inclusion of research subjects.

Exclusion criteria: A total of 3689 male patients and 86 participants with missing or implausible values for key variables. Ultimately, 5984 female patients in T2DM were included in the analysis (**Figure 1**). The follow-up endpoints were death, loss to follow-up, or the end of the follow-up period (December 31, 2024).

Sample representativeness: To ensure the sample is representative of the local female population with type 2 diabetes mellitus (T2DM), no exclusion criteria based on ethnicity or socioeconomic status were used. Data were obtained from the National Basic Public Health Service Management Platform, theoretically covering patients with varying disease severity and stages from diagnosis to recurrence.

### 2.3 Baseline data collection

#### 2.3.1 Questionnaire survey

The questionnaire survey used the Community Diabetes Comprehensive Intervention and Application Project Individual Questionnaire designed by the Jiangsu Provincial Center for Disease Control and Prevention (see Appendix 1), which included general demographic information, health-related behaviors, and disease history. The survey was conducted face-to-face by uniformly trained and qualified personnel. Sleep duration was obtained by asking, “How much time do you usually spend sleeping in a day?” Smoking was defined as having smoked at least 100 cigarettes since starting. Drinking was defined as consuming alcohol at least once a month on average or over 70g pure alcohol per week and not having quit at the time of the survey. High-intensity physical activity was defined as activities requiring significant physical effort, such as lifting heavy objects or plowing, or activities causing a significant increase in breathing and heart rate, lasting at least 10 minutes. Disease duration: the time between the baseline survey date and the date of first diabetes diagnosis.

#### 2.3.2 Physical measurements

Physical measurements included height, weight, and blood pressure. Height measurement required participants removed shoes, hats, and outerwear; women let down their hair, standing upright with knees together and eyes forward, and the reading was taken at eye level with the plate, accurate to 0.1 cm. Weight measurement required participants to wear a single layer of clothing, remove any carried items, and stand in the center of the scale with feet symmetrically placed. Participants stood upright with arms naturally hanging at their sides, heads straight, and eyes looking forward. The weight was recorded when the scale reading stabilized, accurate to 0.1 kg. Blood pressure was measured using an Omron HBP1300 electronic blood pressure monitor. Participants were asked to avoid vigorous exercise or eating for at least one hour before measurement and to rest quietly for more than five minutes. The left elbow was placed flat on the table, with the palm facing up, and the cuff was wrapped from above, with the bottom of the cuff 1–2 cm above the inner elbow. The air tube was aligned with the middle finger. Blood pressure was measured three times, with one-minute intervals between measurements, and the average of the last two readings was calculated.

#### 2.3.3 Laboratory test

The laboratory testing process included on-site sample collection and processing, sample storage and transportation, and blood sample analysis. Blood samples were collected in the morning after fasting(Fasting ≥8 hours), then centrifuged and aliquoted. The aliquoted samples were sent to the Nanjing Jinwei Medical Testing Center for comprehensive analysis of glycated hemoglobin (HbA1c), total cholesterol (TC), triglycerides (TG), high-density lipoprotein cholesterol (HDL-C), low-density lipoprotein cholesterol (LDL-C), alanine aminotransferase (ALT), aspartate aminotransferase (AST),UREA, uric acid (UA), and other related indicators. HbA1c was measured using ion-exchange high-performance liquid chromatography (HPLC), which is certified by the National Glycohemoglobin Standardization Program (NGSP) and the International Federation of Clinical Chemistry and Laboratory Medicine (IFCC). Analysis was performed on a Bio-Rad D-10^®^ automated analyzer (Bio-Rad Laboratories, California, USA).

### 2.4 Index definition

Menopause (8): Women with regular menstrual cycles were defined as premenopausal. Postmenopausal was defined as having no menstruation for at least 12 months (excluding cessation due to medication, pregnancy, severe weight loss, or other pathological reasons).

T2DM (9): Fasting blood glucose ≥7.0 mmol/L or 2-hour postprandial blood glucose ≥11.1 mmol/L, or self-reported history of type 2 diabetes, excluding type 1 diabetes patients. All type 2 diabetes patients in this study were diagnosed by township or community-level hospitals or above. YOD was defined as onset age <40 years, and LOD as onset age ≥40 years.

Hypertension (10): Average blood pressure measured three times, with systolic blood pressure ≥140 mmHg or diastolic blood pressure ≥90 mmHg, or diagnosed with hypertension by a district/county-level hospital or above.

Stroke (11): Diagnosed according to the technical and industry diagnostic standards set by the Chinese Cardiovascular Disease Center or by a district/county-level hospital or above.

Coronary artery disease (12): (1) Diagnosed in accordance with the diagnostic criteria for coronary heart disease (CHD) established by the World Health Organization (WHO); (2) Confirmed by coronary angiography to have ≥50% luminal stenosis in a single major coronary artery or multiple coronary arteries.

Triglyceride glucose-body mass index (TyG-BMI): A derivative of body mass index (BMI) and triglyceride-glucose index (TyG), which captures BMI, blood glucose, and lipid profiles, and better reflects insulin resistance (IR) than TyG alone (13). The formula (14, 15) is: BMI = weight (kg)/height (m²).

TyG = ln [TG (mg/dL) × FPG (mg/dL)/2].

TyG-BMI = TyG × BMI.

### 2.5 Criteria for grouping

Patients were divided into four groups on the basis of whether the onset age was less than 40 years and whether they were postmenopausal:

1. Young menopausal group (YM, n=120)
2. Young premenopausal group (YP, n=267)
3. Non-young menopausal group(NYM, n=5090)
4. Non-young premenopausal group (NYP, n=507)

### 2.6 Ascertainment of mortality

In this study, a rigorous data matching method was adopted to systematically match and cross-validate the basic demographic information (including but not limited to key identifying information such as name, gender, ID number, etc.) of 5,984 enrolled patients with type 2 diabetes who met the study criteria against the Huai’an Resident Death Medical Registration Database (with data updated up to December 31, 2024). Ultimately, the all-cause mortality outcome data of all subjects in the study cohort were successfully obtained and confirmed, including key endpoint indicators such as vital status (alive/deceased) and time of death.

### 2.7 Statistical analysis

Statistical analysis was conducted using SPSS 27.0 and R statistical version 4.3.3.The Kolmogorov–Smirnov test was used to test the normal distribution of each variable. The data are expressed as percentages, medians (25th and 75th percentiles), or means ± standard deviations (SDs). For comparisons between two groups, Student’s t test was used for normally distributed data, and the Mann–Whitney U test was used for nonnormally distributed data. Categorical variables were compared using the chi-square test. For comparisons among four groups, one-way ANOVA was used for normally distributed data with homogeneity of variance, and the Kruskal–Wallis H test was used for nonnormally distributed or heterogeneous data. Categorical data were compared using the chi-square test or Fisher’s exact test. The primary endpoint was all-cause mortality. We compared the baseline general conditions and clinical indicators of women with different ages of onset before and after menopause. Variables with p < 0.1 were selected through univariate COX regression analysis. To control for the influence of confounding factors on the analysis results, the model was stepwise adjusted for the following covariates: systolic blood pressure, diastolic blood pressure, insulin injection, oral medication, UA, sleep duration, diabetes duration, high-intensity activity and stroke, and other known or potential risk factors for mortality. Multivariate Cox regression analysis was used to determine the factors associated with mortality risk in women with different ages of onset before and after menopause. Proportional hazards assumption test: By observing whether the Kaplan-Meier survival curves before and after menopause of women with different ages of onset cross, determine if the proportional hazards assumption is violated. Subgroup analyses were performed to assess potential effect modification by diet control, hypertension, oral medication, and insulin injection. P < 0.05 was considered statistically significant.

## 3 Results

### 3.1 Overall population all-cause mortality analysis

**Figure 2:**
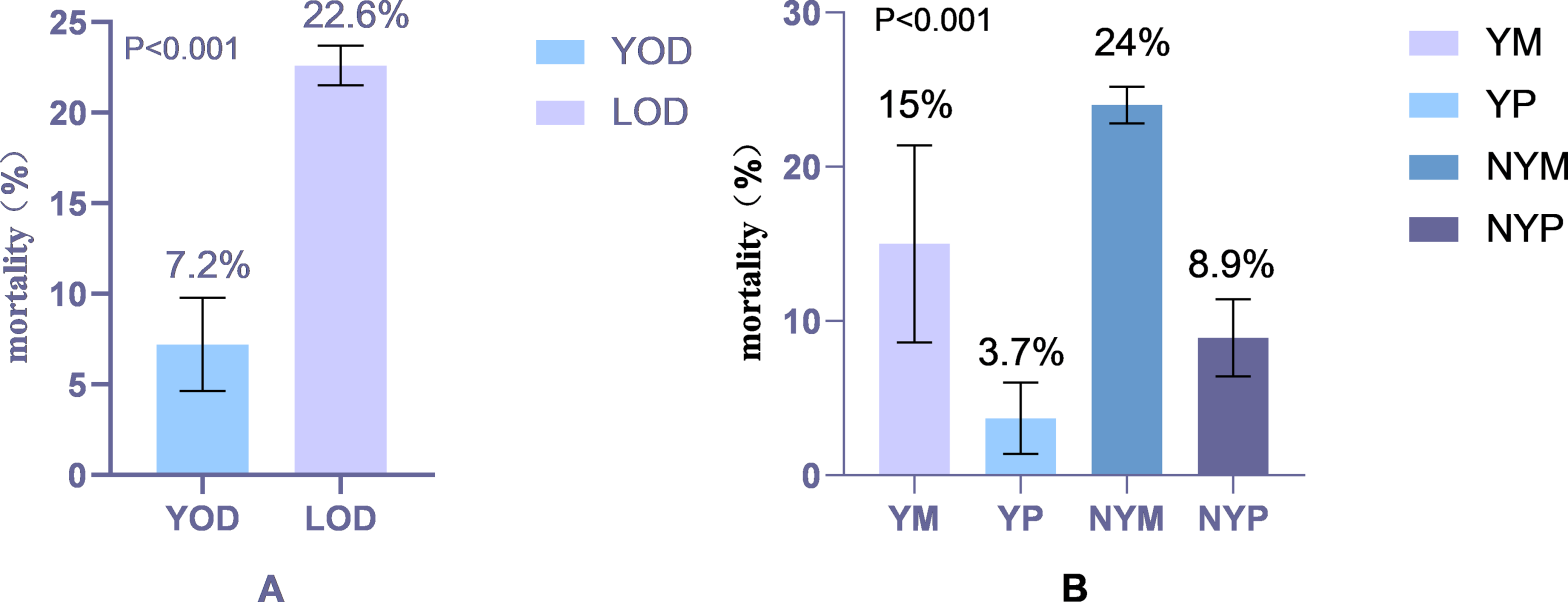
**(A)** Comparison of mortality rates based on different ages of onset. **(B)** Comparison of mortality rates stratified by different ages of onset and menopausal status. (YOD, Young-onset T2DM; LOD, Late-onset T2DM; YM, Young menopausal group; YP, Young premenopausal group; NYM, Non-young menopausal group; NYP, Non-young premenopausal group).

The study population included 5984 female patients with T2DM at baseline, with an average age of 61.77 ± 9.87 years, including 387 YOD patients (6.5%). After follow-up for 10 years, 1293 deaths were recorded, with an all-cause mortality rate of 21.6%. The mortality rate of YOD was lower than that of LOD (7.2% vs. 22.6%, P < 0.001) (**Figure 2A**). However, when divided into four groups, NYM had the highest all-cause mortality rate at 24%, followed by YM at 15%, and YP had the lowest mortality rate at 3.7% (P < 0.001) (**Figure 2B**).

### 3.2 Comparison of general conditions and clinical indicators in women with different ages of onset before and after menopause

To further analyze the clinical characteristics and mortality risk factors for YOD, the general conditions and clinical indicators of women with different ages of onset were compared as follows (**Table 1**):

**Table 1:** Comparison of baseline general conditions and clinical indicators in women with different onset ages.

Clinical data	YOD (n = 387)	LOD (n = 5597)	P
Age (years)	44.00 (40.00, 49.00)	63.00 (57.00, 69.00)	<.001
High school or above,n (%)	39 (10.10)	259 (4.66)	<.001
Smoking,n (%)	4 (1.03)	200 (3.58)	0.008
Drinking,n (%)	28 (7.24)	975 (17.42)	<.001
Sleep duration (min per day)	480.00 (360.00, 490.00)	420.00 (360.00, 480.00)	<.001
High-intensity activity,n (%)	67 (17.31)	750 (13.40)	0.072
Rice,n (%)	386 (99.74)	5570 (99.55)	0.884
Whole grains,n (%)	353 (91.45)	5182 (92.68)	0.370
Age at menarche (years)	16.00 (14.00, 17.00)	17.00 (15.00, 18.00)	<.001
Diabetes duration (years)	18.42 (14.17, 24.50)	13.67 (11.83, 17.25)	<.001
BMI	25.36 (23.30, 27.76)	25.73 (23.43, 28.07)	0.087
Systolic blood pressure (mmHg)	130.50 (120.00, 146.50)	145.00 (131.50, 160.50)	<.001
Diastolic blood pressure (mmHg)	80.00 (74.00, 87.50)	80.00 (73.50, 87.00)	0.762
Laboratory index			
HbA1c (%)	8.10 (6.70, 10.00)	7.20 (6.30, 8.70)	<.001
TC (mmol/L)	5.00 (4.34, 5.77)	5.33 (4.65, 6.07)	<.001
TG (mmol/L)	1.43 (1.04, 2.16)	1.68 (1.20, 2.39)	<.001
LDL-C (mmol/L)	3.08 (2.48, 3.79)	3.32 (2.72, 3.99)	<.001
HDL-C (mmol/L)	1.39 (1.17, 1.67)	1.43 (1.22, 1.72)	0.037
ALT (U/L)	20.00 (16.00, 28.00)	21.00 (16.00, 28.00)	0.424
AST (U/L)	19.00 (16.00, 25.00)	22.00 (18.00, 27.00)	<.001
GGT (U/L)	22.00 (16.00, 31.50)	24.00 (17.00, 35.00)	<.001
Cr (μmol/L)	56.00 (49.00, 64.00)	62.00 (54.00, 73.00)	<.001
UREA (mmol/L)	5.10 (4.20, 6.00)	5.50 (4.60, 6.50)	<.001
UA (μmol/L)	239.00 (199.50, 291.50)	278.00 (233.00, 332.00)	<.001
TyG-BMI	197.88 (173.06, 221.95)	198.82 (175.40, 222.94)	0.603
Baseline Diseases,n (%)			
Stroke	17 (4.39)	771 (13.78)	<.001
Coronary heart disease	27 (6.98)	605 (10.81)	0.047
Hypertension	168 (43.75)	4039 (72.32)	<.001
Diabetes Treatment,n (%)			
Insulin use	101 (26.10)	553 (9.88)	<.001
Oral medication use	251 (64.86)	3790 (67.71)	0.246

Data are expressed as mean (SD), median (interquartile range), or n (%) as appropriate. Student’s t-test or Mann-Whitney U test was used for intergroup comparisons, and the Chi-square (X2) test was used for all categorical count data. BMI, body mass index; HbA1c, glycated hemoglobin; TC, total cholesterol; TG, triglycerides;LDL-C, low-density lipoprotein cholesterol; HDL-C, high-density lipoprotein cholesterol; ALT, alanine aminotransferase; AST, aspartate aminotransferase; Cr, creatinine; UACR, urinary albumin to creatinine ratio;UA, uric acid;TyG-BMI, triglyceride glucose-body mass index.

Compared with LOD, YOD was associated with younger age, higher education, less smoking and alcohol consumption, longer sleep duration, earlier age of menarche, longer duration of diabetes, lower systolic blood pressure, higher HbA1c, and lower levels of TC, TG, LDL-C, HDL-C, AST, GGT, Cr, UREA and UA. Patients with YOD used more insulin to control blood glucose and had fewer cases of coronary stroke, heart disease, and hypertension (P < 0.05).

Interestingly, the all-cause mortality risk of YOD patients increased significantly after menopause. To further analyze the impact of menopause on female T2DM patients with different ages of onset, the patients were divided into four groups based on menopausal status. Compared with the other three groups, the YM group had a longer diabetes duration, higher HbA1c, higher LDL-C, higher UREA, and more people using insulin for diabetes treatment (P < 0.001).

However, compared with the YM patients, the YP patients were younger and had a greater proportion of smokers; longer sleep duration; higher prevalence of hypertension; lower prevalence of stroke; earlier menarche; shorter diabetes duration; and lower SBP, HbA1c, TC, LDL-C, HDL-C, Cr, UREA, and UA. In addition, patients with YP used more insulin to control blood glucose and had fewer cases of coronary stroke, heart disease, and hypertension. However, the patients in NYM were older; had earlier menarche; had a shorter diabetes duration; had higher SBP, TG, AST, GGT, and UA; had lower HbA1c; there were fewer people using insulin for diabetes treatment; and had more hypertension than did those in YM. The patients in the NYP group had more people with higher education levels, longer sleep durations, more people engaging in high-intensity activities, higher TG levels, and shorter durations of diabetes. These patients had lower HbA1c, HDL-C, Cr, and UREA levels and used less insulin and oral hypoglycemic agents for blood glucose control (**Table 2**).

**Table 2:** Comparison of baseline general conditions and clinical indicators in women with different onset ages before and after menopause.

Clinical data	YM (n = 120)	YP (n = 267)	NYM (n = 5090)	NYP (n = 507)	P
Age (years)	52.00 (47.00,57.00)	41.00 (38.00,45.00)^a^	64.00 (58.00,70.00)^a^	49.00 (46.00,53.00)	<.001
High school or above,n (%)	7 (5.83)	32 (12.03)	216 (4.27)	43 (8.51)	<.001
Drinking,n (%)	3 (2.50)	1 (0.37)	189 (3.72)	11 (2.17)	0.009
Smoking,n (%)	16 (13.33)	12 (4.49)^a^	929 (18.25)	46 (9.07)	<.001
Sleep duration (min per day)	420.00 (360.00,480.00)	480.00 (420.00,515.00)^a^	420.00 (360.00,480.00)	480.00 (400.00,480.00)^a^	<.001
High-intensity activity,n (%)	16 (13.33)	51 (19.10)	635 (12.48)	115 (22.68)^a^	<.001
Rice,n (%)	120 (100.00)	266 (99.63)	5065 (99.53)	505 (99.80)	0.939
Whole grains,n (%)	108 (90.76)	245 (91.76)	4718 (92.76)	464 (91.88)	0.694
Age at menarche (years)	16.00 (15.00,18.00)	15.00 (14.00,17.00)^a^	17.00 (16.00,18.00)^a^	16.00 (15.00,18.00)	<.001
Diabetes duration (years)	25.25 (21.23,30.98)	16.33 (13.08,20.12)^a^	13.83 (11.83,17.50)^a^	12.75 (11.75,14.58)^a^	<.001
BMI	25.40 (23.36,27.75)	25.20 (23.30,27.76)	25.72 (23.42,28.05)	25.80 (23.50,28.31)	0.246
Systolic blood pressure (mmHg)	137.25 (124.38,153.50)	127.50 (118.38,141.00)^a^	146.00 (132.50,161.00)^a^	138.00 (126.00,153.00)	<.001
Diastolic blood pressure (mmHg)	80.75 (73.38,88.00)	79.75 (74.00,87.12)	80.00 (73.50,87.00)	81.50 (75.00,89.00)	0.005
Laboratory index					
HbA1c (%)	8.40 (7.10,9.72)	7.90 (6.45,10.05)^a^	7.30 (6.30,8.70)^a^	7.00 (6.00,8.70)^a^	<.001
TC(mmol/L)	5.30 (4.70,6.04)	4.81 (4.23,5.59)^a^	5.34 (4.67,6.08)	5.08 (4.40,5.86)	<.001
TG(mmol/L)	1.32 (1.02,2.05)	1.49 (1.04,2.23)	1.70 (1.21,2.41)^a^	1.56 (1.13,2.14)^a^	<.001
LDL-C(mmol/L)	3.38 (2.69,3.90)	2.96 (2.42,3.66)^a^	3.34 (2.72,4.00)	3.13 (2.64,3.87)	<.001
HDL-C(mmol/L)	1.50 (1.25,1.87)	1.33 (1.14,1.60)^a^	1.44 (1.22,1.72)	1.39 (1.19,1.66)^a^	<.001
ALT(U/L)	19.00 (16.00,28.00)	20.00 (16.00,29.00)	21.00 (16.00,28.00)	21.00 (17.00,28.00)	0.789
AST(U/L)	20.00 (17.00,25.25)	19.00 (16.00,24.50)	22.00 (18.00,27.00)^a^	21.00 (17.00,25.00)	<.001
GGT(U/L)	22.00 (15.75,31.25)	22.00 (16.00,31.50)	24.00 (17.00,35.00)^a^	23.00 (16.00,34.00)	<.001
Cr(μmol/L)	61.00 (52.75,70.00)	54.00 (48.00,62.00)^a^	63.00 (55.00,73.00)	58.00 (50.00,65.00)^a^	<.001
UREA(mmol/L)	5.55 (4.65,6.70)	4.90 (4.00,5.80)^a^	5.50 (4.60,6.60)	5.10 (4.20,6.10)^a^	<.001
UA(μmol/L)	245.50 (211.50,301.50)	237.00 (194.50,281.00)^a^	280.00 (236.00,334.00)^a^	252.00 (212.00,296.50)	<.001
TyG-BMI	193.62 (173.87,221.63)	200.35 (173.06,221.95)	199.13 (175.58,222.55)	195.78 (173.92,224.16)	0.803
Baseline Diseases,n (%)					
Stroke	9 (7.50)	8 (3.00)^a^	739 (14.52)	32 (6.31)	<.001
Coronary heart disease	13 (10.83)	14 (5.24)^a^	580 (11.39)	25 (4.93)^a^	<.001
Hypertension	69 (57.50)	99 (37.50)^a^	3757 (73.99)^a^	282 (55.62)	<.001
Diabetes Treatment,n (%)					
Insulin use	46 (38.33)	55 (20.60)^a^	516 (10.14)^a^	37 (7.30)^a^	<.001
Oral medication use	81 (67.50)	170 (63.67)	3502 (68.80)	288 (56.80)^a^	<.001

a indicates comparison with the YM group P < 0.05. BMI, body mass index; HbA1c, glycated hemoglobin; TC, total cholesterol; TG, triglycerides; LDL-C, low-density lipoprotein cholesterol; HDL-C, high-density lipoprotein cholesterol; ALT, alanine aminotransferase; AST, aspartate aminotransferase; Cr, creatinine; UACR, urinary albumin to creatinine ratio; UA, uric acid;TyG-BMI, triglyceride glucose-body mass index.

### 3.3 Survival curves for women of different ages at onset with or without menopause

**Figure 3:**
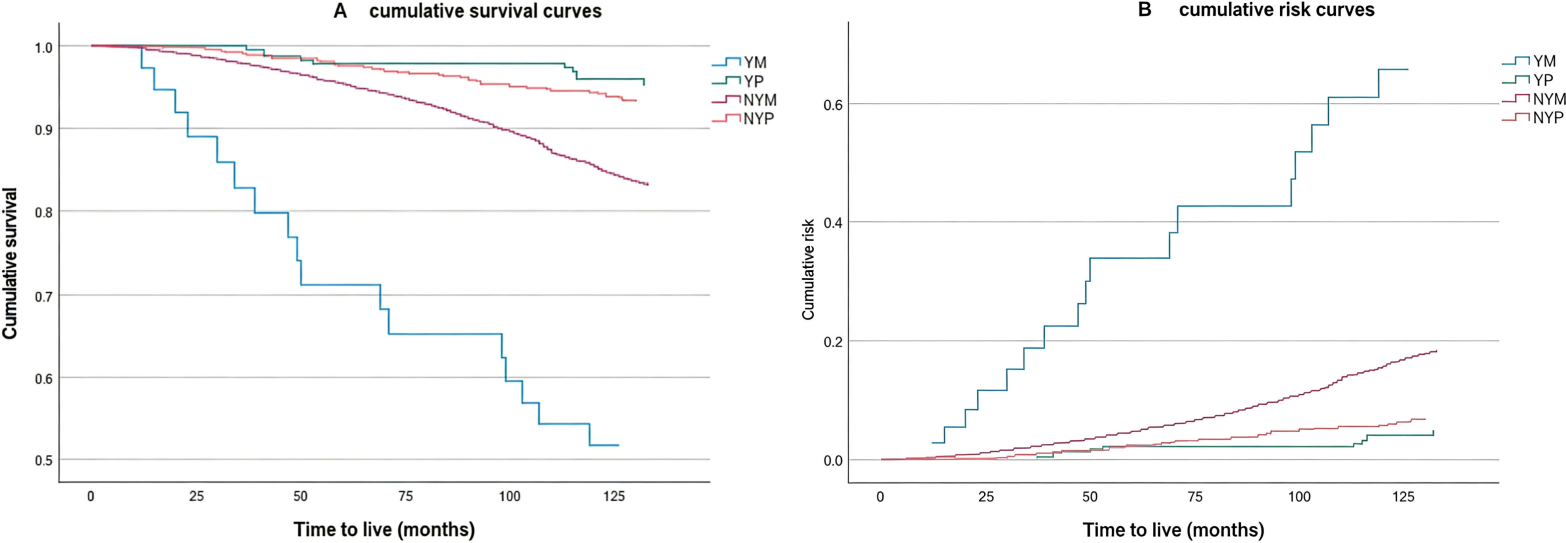
Cumulative survival curves **(A)** and cumulative risk curves **(B)** based on age at onset and menopausal status. (YM, Early-onset T2DM postmenopausal group; YP, Early-onset T2DM premenopausal group; NYM, Late-onset T2DM postmenopausal group; NYP, Late-onset T2DM premenopausal group).

To explore the associations between the onset age of T2DM, menopausal status, and all-cause mortality risk, cumulative survival curves and risk curves were plotted after adjustment for confounders including systolic blood pressure, diastolic blood pressure, insulin injection, oral medication, BUN, UA, sleep duration, diabetes duration, high-intensity activity and stroke (**Figure 3**). The results revealed that YM had the lowest survival curve, followed by NYM, and YP had the highest survival curve (**Figure 3A**). The study also revealed that YM had the highest risk curve, whereas YP had the lowest risk curve (**Figure 3B**).

### 3.4 Analysis of mortality risk factors in women with different onset ages with and without menopause

To further compare and analyze the factors influencing mortality risk in women with different ages of onset after menopause, multivariate Cox regression analysis was performed for each group (**Table 3**). The results revealed that the TyG-BMI was an independent protective factor for YM (P = 0.018). For every one standard deviation increase in the TyG-BMI, the mortality risk decreased by 2.0%. UA was a common risk factor in women with different ages of onset with or without menopause.

**Table 3:** Analysis of mortality risk factors in women with different onset ages before and after menopause.

Variable	YM (n=120)		YP (n=267)		NYM (n=5090)		NYP (n=507)	
	HR (95%Cl)	P	HR (95%Cl)	P	HR (95%Cl)	P	HR (95%Cl)	P
TyGBMI	0.980 (0.965,0.997)	0.018	–	–	–	–	–	–
UA	1.007 (1.001,1.013)	0.022	1.010 (1.003,1.017)	0.004	1.002 (1.001,1.002)	<0.001	1.004 (1.000,1.007)	0.032
Oral medication use	–	–	–	–	0.590 (0.518,0.672)	<0.001	0.209 (0.094,0.467)	<0.001
Insulin use	–	–	–	–	0.285 (0.244,0.333)	<0.001	0.219 (0.082,0.585)	0.002
SBP	–	–	1.058 (1.023,1.095)	0.001	1.014 (1.011,1.017)	<0.001	1.032 (1.015,1.050)	<0.001
DBP	–	–	0.916 (0.840,0.999)	0.048	0.976 (0.970,0.982)	<0.001	–	–
Sleep duration	–	–	–	–	–	–	–	–
Diabetes duration	–	–	0.699 (0.544,0.899)	0.005	0.792 (0.778,0.807)	<0.001	0.711 (0.629,0.805)	<0.001
High-intensity activity	–	–	–	–	1.643 (1.477,1.827)	<0.001	2.736 (1.622,4.615)	<0.001
Stroke	–	–	–	–	–	–	–	–

Variables not listed in the table with hazard ratio (HR) values and 95% confidence intervals had no significant impact on mortality risk (p > 0.05).

### 3.5 Forest plot of subgroup analysis between TyG-BMI index and YM all-cause mortality

**Figure 4:**
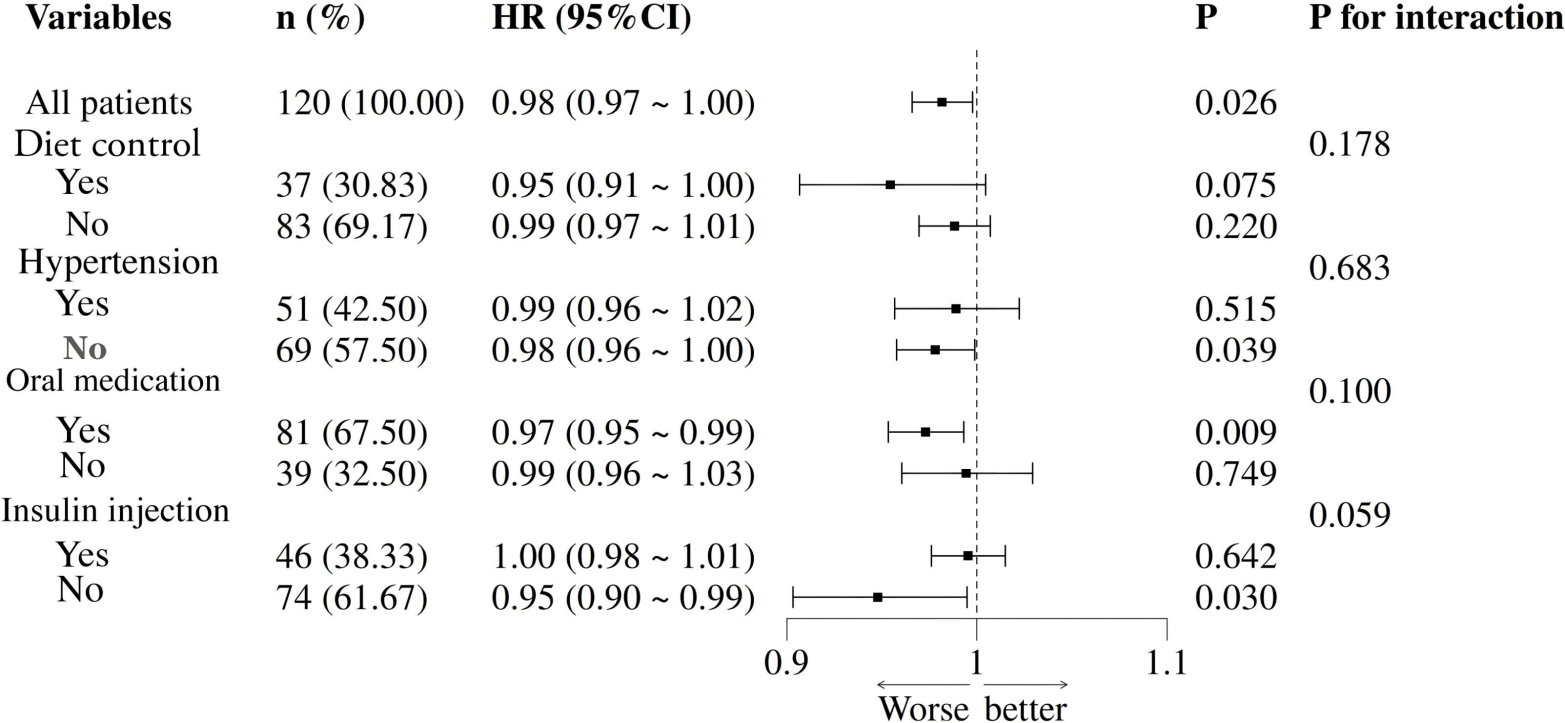
Forest plot of subgroup analysis between TyG-BMI index and YM all-cause mortality. Forest plot of subgroup analysis between TyG-BMI index and YM all-cause mortality. This forest plot presents the hazard ratios (HR) and 95% confidence intervals (CI) derived from an adjusted covariate Cox proportional hazards model. The included variables are sleep duration, age at menarche, diabetes duration, SBP, DBP, and UA. The reference line is HR = 1.

The results of the survival analysis of different subgroups in different age-at-onset groups in this study further showed that the protective effect of TyG-BMI was consistent across subgroups of dietary control, hypertension, oral medication, and insulin injection (interaction P value > 0.05) (**Figure 4**). However, the association between TyG-BMI and all-cause mortality of YM was stronger in patients with baseline dietary control and those not using insulin injection (interaction P value < 0.05).

In the YM group, patients without hypertension at baseline had a 2% lower risk of death; patients taking oral antidiabetic drugs at baseline had a 3% lower risk of death; and patients not using insulin injections at baseline had a 5% lower risk of death.

## 4 Discussion

YOD is becoming increasingly common and significantly impacts disease burden. According to data from the 2019 Global Burden of Disease Study, the burden of YOD clearly increased from 1990–2019 (16). There are few epidemiological data on female YOD. A total of 5984 women with T2DM were included in the study, including 387 with YOD, accounting for 6.5% of those diagnosed with T2DM. After 10 years of follow-up, there were 1293 all-cause deaths, with an overall all-cause mortality rate of 21.6%. The mortality rate of YOD patients was lower than that of LOD patients (7.2% vs. 22.6%) among women. However, when divided into four groups based on onset age and menopausal status, the all-cause mortality risk of YOD patients increased significantly after menopause. This study revealed that the mortality risk of YOD was influenced by menopausal status. Subsequent subgroup analysis of the four groups revealed that the TyG-BMI was an independent protective factor for all-cause deaths in the YM cohort. For every one standard deviation increase in TyG-BMI, the mortality risk decreased by 2.0% in YOD with menopause.

Multiple interrelated biological mechanisms may explain the association between elevated TyG-BMI and reduced risk of YM mortality. Higher TyG-BMI values likely indicate enhanced substrate energy availability during acute catabolic stress. In critical illness, the body prioritizes gluconeogenesis and lipolysis to meet increased energy demands (17). Elevated fasting triglycerides and glucose (components of the TyG index) may provide a readily mobilizable substrate for ATP generation in vital organs, particularly the heart. Adipose tissue-derived free fatty acids (FFAs) serve as an important energy source for myocardial cells under stress (18), while hepatic glycogenolysis maintains systemic glucose availability (19). Beyond energy storage, adipose tissue has pleiotropic functions. In acute illness, visceral fat may act as a ‘sink’ for pro-inflammatory cytokines (20).Adipose tissue may also buffer inflammatory mediators and provide endocrine protection during acute diseases (21).For critically ill patients, a higher TyG-BMI may reflect better nutritional reserves and metabolic status, while a lower TyG-BMI may indicate malnutrition or cachexia. High-risk patients with low TyG-BMI should be identified early and provided with enhanced monitoring and nutritional support.

The prevalence of YOD in the general population in China is approximately 3.5%, and the prevalence of YOD in the population of patients diagnosed with diabetes is 10%~15% (22). However, this study revealed that the prevalence of YOD among patients diagnosed with T2DM was 6.5%, which was lower than the national level. Possible reasons are as follows. First, this study was conducted in the economically developed Jiangsu region, where the Huaiyang cuisine is light and diverse (23). Second, the patients in this study were all registered and enrolled in the National Basic Public Health Service Management Program in the Qinghe District (now Qingjiangpu District) and Huai’an District of Huai’an city, Jiangsu Province, where the health care conditions are better and community screening and intervention capabilities are stronger (24).

Ji Linong’s team reported that, compared with type 1 diabetes (T1DM) patients with similar onset ages, YOD patients (15–30 years) had significantly higher rates of nephropathy, neuropathy, macrovascular complications, and cardiovascular adverse events, with a higher mortality rate (11%) (25). However, after 10 years of follow-up, the all-cause mortality rate was 21.6% in the female cohort. The mortality rate of female YOD patients was 7.2%, which was not only lower than the overall YOD population all-cause mortality rate (11%) reported in the literature but also lower than the LOD mortality rate (22.6%) in this cohort. This contradicts the literature. Huo et al. (26) believe that the risk of nonfatal cardiovascular disease in YOD increased with prolonged exposure to hyperglycemia and worsening metabolic environments and was 1.91-fold greater than that of the LOD. Compared with LOD patients, YOD patients have more rapid β-cell function decline, faster progression of diabetes complications, and a higher prevalence of obesity and comorbidities (1, 27). YOD increases mortality risk, and that this is mainly through earlier CVD mortality (28).YOD patients have a relatively longer duration of diabetes. We believe that a longer duration of diabetes was the most fundamental driving factor leading to poorer prognosis in the YOD group. However, when the patients were divided into four groups based on menopausal status, the mortality rate of postmenopausal YOD patients was higher than that of premenopausal LOD patients, which was similar to the 11% reported in the literature (25). After adjusting for confounding factors such as TyG-BMI, systolic blood pressure, diastolic blood pressure, insulin injection, oral medication, BUN, UA, sleep duration, diabetes duration, high-intensity activity and stroke, cumulative survival curves and risk curves were plotted. Compared with premenopausal YOD and LOD populations, postmenopausal YOD populations presented the highest risk curve and the lowest survival curve. Therefore, the all-cause mortality risk of female YOD patients has unique characteristics and is influenced by menopausal status. Moreover, the impact of menopausal status on the mortality risk of YOD patients far exceeded that on the LOD mortality risk.

Why does menopause have such a significant effect on the mortality risk of YOD? There is limited literature on this topic. To further analyze the clinical characteristics and mortality risk factors for YOD patients, subgroup analyses were conducted on the mortality risk factors in the four groups. The results showed that the TyG-BMI was the only independent protective factor for all-cause mortality risk in postmenopausal YOD patients. As the TyG-BMI increased, the mortality risk significantly decreased. The TyG-BMI has gradually gained attention in the fields of metabolic syndrome, insulin resistance, and cardiovascular disease research (29). According to a recent study, the TyG-BMI can capture multiple clinical variables, such as BMI, blood glucose, and lipid levels, and better reflects insulin resistance (IR) than can the index alone (15). Similar results have been reported in previous studies (30), where for every one standard deviation increase in the TyG-BMI in diabetes patients, the corresponding all-cause mortality and cardiovascular disease mortality decreased by 23% (HR 0.77, 95% CI 0.69–0.86) and 36%(HR 0.64, 95% CI 0.48-0.86), respectively. In this study, the TyG-BMI in the postmenopausal YOD group was significantly lower than that in the other groups. After adjusting for confounding factors, for every one standard deviation increase in TyG-BMI, the all-cause mortality risk in postmenopausal YOD women decreased by 2.0%. Therefore, we speculated that the impact of menopause on YOD mortality risk was also achieved through complex metabolic state changes affecting the development of cardiovascular diseases. However, the risk reduction in the postmenopausal YOD group was lower than the mortality risk reported in the literature (2.0% vs. 23%). First, sex differences may affect mortality risk. The study subjects in this study were all women. Research (31) has shown that the relative risk of cardiovascular events in female diabetes patients is greater than that in male diabetes patients and that the risk of coronary artery disease (CAD), the leading cause of death in postmenopausal women, is greater (32). Second, the relative risks of cardiovascular disease mortality and all-cause mortality differ among T2DM patients with different ages of onset, which is more pronounced in YOD (33). Considering the above points, the pathogenesis of postmenopausal YOD patients is complex, with a relatively high mortality risk. Therefore, even with changes in metabolic state (reducing TyG-BMI levels), the degree of risk reduction in this group remains relatively low.

However, a two-year study (34) involving 200 women aged 45–60 years reported that the incidence of metabolic syndrome in the entire study group was 29%. The incidence of metabolic syndrome in the premenopausal group was 16%, whereas in the postmenopausal group, it was 42%. Possible reasons include that postmenopausal women are more prone to metabolic syndrome due to estrogen deficiency and fat redistribution and are more likely to experience physical discomfort. Vasomotor symptoms are positively correlated with metabolic syndrome (35). Therefore, we infer that YOD patients are more likely to develop metabolic syndrome after menopause, leading to poor blood glucose control, the activation of oxidative stress and inflammation, and the production of vascular endothelial growth factors, which may accelerate the progression of diabetes microvascular complications and increase all-cause mortality risk. Therefore, the TyG-BMI remains a very economical and convenient index for reflecting all-cause mortality risk in postmenopausal YOD women and is more suitable for large-scale epidemiological studies and clinical screening in China, further guiding the early identification and intervention of high-risk female populations.

We observed that the impact of TyG-BMI on clinical outcomes was highly dependent on the patients’ treatment regimen. In patients treated with oral glucose-lowering medications only, higher TyG-BMI may be associated with a lower risk of mortality. However, this association disappears in patients requiring insulin therapy. This suggests that treatment regimen must be considered when evaluating the prognostic value of biomarkers.

This study also revealed that UA was a common risk factor for all-cause mortality risk in women with different ages of onset before and after menopause. The observed relationship between UA levels and increased mortality is biologically plausible and may involve vertebral mesothelial dysfunction, cardiometabolic syndrome, and inflammation. Uric acid tends to precipitate into crystals, and urate is deposited on the walls of blood vessels, leading to reduced levels of nitric oxide and nitric oxide in vascular endothelial dysfunction, which may promote atherosclerosis and thrombosis (36). Previous studies have also shown a relationship between higher SUA levels and dyslipidemia, including higher triglyceride levels and lower HDL cholesterol levels, which are significant risk factors for cardiovascular health (37). Hyperuricemia increases the mortality of women with coronary heart disease, and more significantly than that of men (38). We consider the possible reason to be the potential cardioprotective effect of estrogen in women (39–41). However, in postmenopausal women, the protective effect is weakened due to decreased estrogen levels, thereby exacerbating the harmful effects of uric acid on women. In addition, female patients with hyperuricemia often present with more severe metabolic disturbances, including higher mean age and blood glucose levels (42).

The main innovations of this study are as follows: to our knowledge, this is the first study to comprehensively analyze all-cause mortality risk and its influencing factors in female YOD patients. Second, the study has a large sample size, multiple confounding factors, and scientific statistical methods, making the results highly reliable. Finally, this study combines cross-sectional and longitudinal data analysis, reflecting the dynamic evolution process, with novel conclusions that enrich the clinical epidemiology of YOD and have significant clinical guidance value.

This study has several limitations. First, it was conducted in only one medical center in Huai’an city, Jiangsu Province, and further research in different regions and ethnic groups is needed to verify the reliability of the results. Second, sample size imbalance may exert certain impacts on statistical power, the accuracy of parameter estimation, and the robustness of the results. Third, more assessment methods, such as female hormone and gynecological ultrasound, were not included. Fourth, some data obtained through self-reports may have reduced accuracy due to recall errors. Fifth, this study relies on baseline TyG-BMI data from a single time point. Due to the lack of repeated measurements of TyG-BMI and long-term follow-up, we are unable to evaluate the natural fluctuations, progression trajectories, or the predictive value of changes in this indicator for clinical outcomes. This may limit our comprehensive understanding of its long-term risk characterization capabilities. Finally, an assessment of diabetes complications was lacking after ten years of follow-up, which may make it more difficult to further analyze the causes of death and explore the underlying mechanisms involved.

## 5 Conclusion

In this study, the overall all-cause mortality rate for YOD was lower than that for LOD. However, postmenopausal women with YOD have greater all-cause mortality than premenopausal women with LOD do, and their risk of death decreases with increasing TyG-BMI. Therefore, early identification and intervention of TyG-BMI levels in postmenopausal women with YOD can effectively improve their mortality risk. Further research is needed in the future to determine if interventions focused on the TyG-BMI index can enhance clinical outcomes in YOD female patients.

## Data Availability

The original contributions presented in the study are included in the article/supplementary material. Further inquiries can be directed to the corresponding authors.
