# Correction: Analysis of mortality risk and influencing factors in female patients with young-onset type 2 diabetes

**DOI:** 10.3389/fendo.2026.1898962

**Published:** 2026-07-02

**Authors:** Ling Xiang, Mingmin Zhu, Zi Yan, Zhijin Geng, Yuankai Zhou, Zhongming Sun, Dandan Miao, Yong Zhang, Jinyi Zhou, Enchun Pan, Wen Hu

**Affiliations:** 1 The Affiliated Huai’an Hospital of Xuzhou Medical University/The Second People’s Hospital of Huai’an, Huai’an, Jiangsu, China; 2 Department of Endocrinology, Sheyang County People’s Hospital, Yancheng, Jiangsu, China; 3 Huai’an Center for Disease Control and Prevention, Huai’an, Jiangsu, China; 4 Jiangsu Provincial Center for Disease Control and Prevention, Nanjing, China

**Keywords:** all-cause mortality, Chinese patients, risk factors, TyG-BMI, young-onset type 2 diabetes mellitus, female patients

Author Jinyi Zhou was erroneously assigned to affiliation “Huai’an Center for Disease Control and Prevention, Huai’an, Jiangsu, China”. This affiliation has now been removed for author Jinyi Zhou. Affiliation “Jiangsu Provincial Center for Disease Control and Prevention” was omitted for author Jinyi Zhou. This affiliation has now been added for author Jinyi Zhou.

Affiliation “The Affiliated Huai’an Hospital of Xuzhou Medical University/The Second People’s Hospital of Huai’an” was erroneously given as “Department of Endocrinology, Huai’an Hospital” Affiliated to “Xuzhou Medical University and Huai’an Second People’s Hospital, Huai’an, Jiangsu, China”.

The original version of this article has been updated.

